# Transposition of Reversed *Ac* Element Ends Generates Novel Chimeric Genes in Maize

**DOI:** 10.1371/journal.pgen.0020164

**Published:** 2006-10-06

**Authors:** Jianbo Zhang, Feng Zhang, Thomas Peterson

**Affiliations:** 1 Genetics, Development, and Cell Biology, Iowa State University, Ames, Iowa, United States of America; 2 Department of Plant Biology, University of Georgia, Athens, Georgia, United States of America; Fred Hutchinson Cancer Research Center, United States of America

## Abstract

The maize *Activator/Dissociation (Ac/Ds)* elements are members of the *hAT (hobo, Ac,* and *Tam3)* superfamily of type II (DNA) transposons that transpose through a “cut-and-paste” mechanism. Previously, we reported that a pair of *Ac* ends in reversed orientation is capable of undergoing alternative transposition reactions that can generate large-scale chromosomal rearrangements, including deletions and inversions. We show here that rearrangements induced by reversed *Ac* ends transposition can join the coding and regulatory sequences of two linked paralogous genes to generate a series of chimeric genes, some of which are functional. To our knowledge, this is the first report demonstrating that alternative transposition reactions can recombine gene segments, leading to the creation of new genes.

## Synopsis

Transposable elements, or “jumping genes,” are DNA segments that can move to new sites in the genome. One type of transposable element from maize, called *Ac/Ds,* moves by a reaction known as “cut-and-paste.” In this mechanism, a transposase enzyme cleaves at both ends of a single *Ac*/*Ds* element, releasing the element from one site and inserting it at another location. However, if two *Ac/Ds* elements are situated near each other, the transposase may sometimes cut at the ends of two different elements. When these two *Ac/Ds* ends insert at a new location, a large rearrangement of the genome can occur; this process is termed alternative transposition. In this work, the authors studied alternative transposition events that affect the structure and expression of two genes that control maize kernel color. Alternative *Ac* transposition can cause fusions of the coding sequences of the two genes, generating a new functional chimeric gene that specifies a new maize kernel color. This mechanism of gene creation through alternative transposition is similar to the way that functional antibody genes are generated in the vertebrate immune system. These results show how the actions of transposable elements can reshuffle the genome to generate new functional genes.

## Introduction

The maize *Ac* element is 4,565 base pairs (bp) in length and encodes an 807–amino acid transposase that catalyzes *Ac/Ds* transposition. The *Ac/Ds* element ends are delineated by complementary 11-bp terminal inverted repeat sequences, while the sub-terminal sequences are distinct from each other [1]. The individual *Ac* termini are designated as 5′ or 3′ according to their proximity to the beginning and end of the *Ac* transcript. Transposition requires one *Ac* 5′ end and one *Ac* 3′ end [2]. In standard transposition, the *Ac* 5′ and 3′ ends are part of a single transposon, and the outcome of transposition is the excision of the element from a donor site and insertion into a target site. However, transposition reactions can also involve the 5′ and 3′ ends of different *Ac/Ds* elements, which can be in either a direct or reversed orientation with respect to each other [3,4]. These alternative transposition events can generate deletions, duplications, inversions, and other sequence rearrangements. Because *Ac/Ds* preferentially transposes into genic regions, the rearrangements induced by alternative *Ac/Ds* transposition would be predicted to shuffle coding and regulatory sequences, and thereby generate new genes. We searched for such events in maize stocks containing a pair of reversed *Ac* ends in the *p1* gene, which regulates kernel pericarp pigmentation. We obtained four chimeric alleles in which the promoter, exon 1 and exon 2 of the *p2* gene (a paralog of *p1*) [5] is joined with exon 3 of the *p1* gene. Because the *p1* and *p2* coding sequences are very similar, the new chimeric genes would encode proteins nearly identical to that encoded by the *p1* gene. The *p2* promoter is inactive in pericarp in the progenitor allele; however, these four new alleles show significant expression in kernel pericarp, and specify a novel orange pericarp phenotype. We propose that this new phenotype is largely caused by an altered expression pattern resulting from the chromosomal rearrangement. These results demonstrate that alternative transposition reactions can generate gene fusions and therefore may have been an important force in gene and genome evolution.

## Results

### Structures of Novel Chimeric Alleles

**Figure 1:**
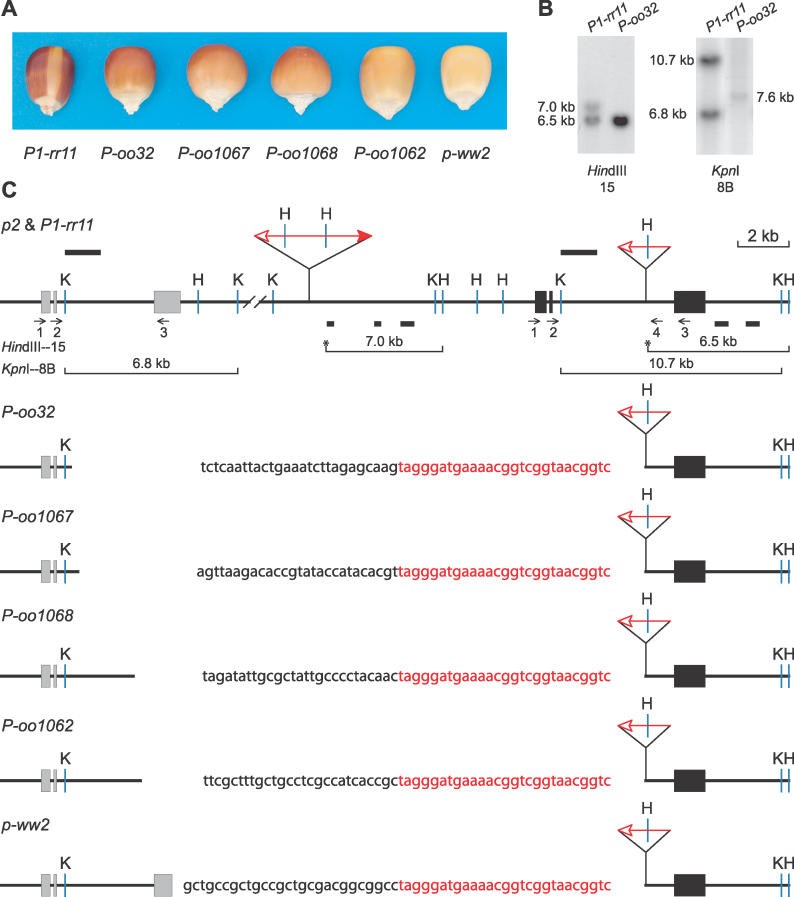
Phenotypes and Gene Structures of *P-oo* Alleles (A) The kernel pericarp pigmentation phenotypes specified by the indicated alleles. (B) Genomic Southern blot. Genomic DNA from plants homozygous for the indicated alleles was cut with KpnI and HindIII, and hybridized with probes 15 or 8B from the *p1* gene. Lanes marked *P-oo32* contain approximately twice as much DNA as lanes marked *P1-rr11;* this DNA overloading enables the detection of the 7.6-kb band in the KpnI 8B blot, but also results in the intense 6.5-kb band in the HindIII 15 blot. (C) Restriction map. The solid and gray boxes are exons 1, 2, and 3 (left to right) of *p1* and *p2,* respectively. Red triangles indicate *Ac* or *fAc* insertions, and the open and solid arrowheads indicate the 3′ and 5′ ends, respectively, of *Ac/fAc*. Sequences hybridizing with Southern blot probes are indicated by the solid bars above (probe 8B) and below (probe 15) the map. The short horizontal arrows indicate the orientations and approximate position of PCR primers. Primers are identified by numbers below the arrows. The sequence of the junction of each fusion allele is shown here; the black letters indicate *p2* sequence, while the red letters indicate *fAc* sequence. K, KpnI; H, HindIII. Lines below the map indicate the restriction fragments produced by digestion with KpnI or HindIII and hybridizing with the indicated probe; asterisks indicate HindIII restriction sites located within *Ac* or *fAc* sequences.

The maize *p1* gene encodes a Myb-homologous transcriptional regulator required for synthesis of red pigments in kernel pericarp (Figure 1) and cob glumes [6]. The *P1-rr11* allele (*r*ed pericarp, *r*ed cob) contains a truncated *Ac* element *(fAc, fractured Ac)* inserted in the second intron of *p1,* and a full-length *Ac* element inserted 13,175 bp upstream of the *fAc* element; the 5′ end of *Ac* and the 3′ end of *fAc* in *P1-rr11* are oriented towards each other (Figure 1C). A paralog of *p1,* termed *p2,* is located approximately 60 kilobases (kb) upstream of the *p1* gene in the chromosome containing the *P1-rr11* allele [7] (Figure 1C). The *p2* gene is not expressed in kernel pericarp and hence does not contribute to pericarp color [5,8]. Reversed *Ac* ends transposition in *P1-rr11* would eliminate *p1* gene function, and most mutants derived from *P1-rr11* have colorless kernel pericarp and cob. However, we did isolate four alleles with orange pericarp and orange cob, and these were designated as *P-oo32, P-oo1062, P-oo1067,* and *P-oo1068* (Figure 1A).

Genetic tests indicate that there is no *Ac* activity in the genome of the *P-oo* alleles. We characterized the structural rearrangements in the *P-oo* alleles by genomic DNA gel blot and PCR. Genomic DNA from plants carrying the *P-oo32* allele was cut with HindIII and KpnI, and hybridized with maize *p1* genomic probe fragments 15 or 8B (Figure 1B). In comparison to *P1-rr11,* the *P-oo32* allele lacks the 7.0-kb HindIII fragment (from *p1*), the 10.7-kb KpnI fragment (from *p1*), and the 6.8-kb KpnI fragment (from *p2*). The absence of these fragments and the lack of *Ac* activity in the genome suggest that *P-oo32* has a deletion that includes both *p1* and *p2* sequences. On the other hand, the presence of the 6.5-kb HindIII fragment detected by *p1* fragment 15 indicates that the 3′ portion of the *p1* gene and at least a part of *fAc* are intact. The faint 7.6-kb fragment in the KpnI-8B blot suggests that the upstream deletion end point is within the 8B-homologous fragment in *p2*. To test this, we performed PCR analysis using oligonucleotide primers 2 and 4, which flank the *fAc* insertion in *p1* and are complementary to corresponding sites in the *p2* gene. A ~2.4-kb product was amplified from *P-oo32* DNA and sequenced. The results indicate that the 3′ end of *fAc* is inserted into a site in intron 2 of *p2* (position 5619 in GenBank sequence AF210616), while the sequence downstream of *fAc* is from intron 2 of *p1* (the *p1* and *p2* sequences are highly homologous, but sufficient sequence polymorphisms exist to distinguish the origin of PCR products). This result, together with the DNA gel blot results, indicates that *P-oo32* is a gene fusion containing exon 1 and exon 2 of the *p2* gene, the *fAc* sequence, and exon 3 of the *p1* gene.

We characterized three additional *P-oo* alleles derived from *P1-rr11: P-oo1062, P-oo1067,* and *P-oo1068*. We performed PCR using primer 4 of the *p1* gene, and a series of primers complementary to intron 2 of *p2*. Sequencing of the PCR products revealed that these three alleles have structures resembling that of *P-oo32*: each has exons 1 and 2 from *p2,* and exon 3 from *p1*. However, each allele exhibits a distinct site of *fAc* insertion in *p2* intron 2: nucleotides 5912, 8088, and 8365 of AF210616 in *P-oo1067, P-oo1068,* and *P-oo1062,* respectively (Figure 1C). Importantly, the rearrangements show precise junctions of the *p2* sequence with the *fAc* terminus. In contrast, transposition of *Ds* elements in *Arabidopsis* is reported to generate large deletions, but the deleted sequences extend into the *Ds* termini, indicating the involvement of cellular DNA repair mechanisms in deletion formation [9]. The precise junctions observed in the *P-oo* alleles are consistent with their formation through a single transposase-mediated insertion event.

### The *P-oo* Alleles Are Generated by Reversed *Ac* Ends Transposition

**Figure 2:**
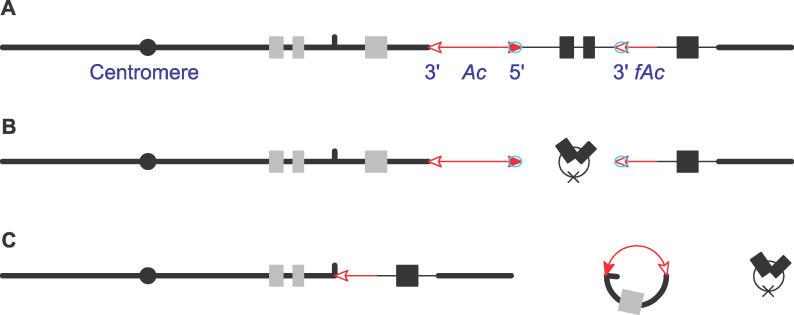
Deletions by Reversed *Ac* Ends Transposition Generate Chimerical Genes The solid circle indicates the centromere, the short vertical line indicates the target site, and the other symbols have the same meaning as those in Figure 1. (For animated version, see Video S1). (A) Ac transposase (blue oval) binds to the 5′ end of *Ac* and 3′ end of *fAc*. (B) As in ordinary transposition, the *Ac* 5′ end and the *fAc* 3′ end are excised by transposase cleavage, and the sequences flanking the *Ac/fAc* ends join together to form a ~13-kb circle. The X mark at the junction indicates the transposon footprint. (C) The excised transposon ends insert into a site in intron 2 of *p2*. The *Ac* 5′ end joins to the distal side of the insertion site to form a circle, and the *fAc* 3′ end joins to the proximal side of the insertion site to generate a chimeric gene containing exon 1 and exon 2 of *p2* and exon 3 of *p1*. This study reports the isolation of the progenitor (A) and deletion products (C). Note that the hypothetical structures shown in (B) are transient in nature and would not be amenable to physical isolation.

As mentioned above, the *p2* gene is located approximately 60 kb proximal to *p1,* and in the same transcriptional orientation [7]. The *P1-rr11* allele contains reverse-oriented *Ac* 5′ and *fAc* 3′ ends whose transposition can generate a variety of chromosomal rearrangements [3]. If the excised *Ac/fAc* ends insert into a site in intron 2 of *p2,* the *fAc* in *p1* intron 2 will be precisely joined to the insertion point in intron 2 of *p2,* and the ~60 kb of DNA between them will be deleted (Video S1). The resulting chromosome will carry a new fusion gene, composed of the promoter, exon 1 and exon 2 of *p2*, joined through *fAc* to exon 3 of *p1* (Figure 2)*.* The structures of *P-oo32, P-oo1062, P-oo1067,* and *P-oo1068* are consistent with their origin via this reversed *Ac* ends transposition mechanism.

We considered an alternative mechanism for generation of the fusion alleles via transposon-induced homologous recombination. Transposition of *Ac/Ds* elements is known to induce recombination between flanking homologous sequences [10,11]. The second introns of the *p1* and *p2* genes are 4.6 and 3.8 kb, respectively, and are 84% identical over their common lengths. Alleles formed by homologous recombination should have crossover sites at homologous sequences. However, the *P-oo* alleles have breakpoints at various sites within the *p2* intron, and each junction occurs precisely at the *fAc* 3′ end. Moreover, all alleles retain the *fAc* sequence, with the sequences upstream of *fAc* resembling *p2* and the sequences downstream of *fAc* resembling *p1.* This structure would not be expected from homologous recombination, but is consistent with transposition-induced rearrangement.

### Expression of the *P-oo* Alleles in Pericarp

In addition to the *P-oo* alleles described above, we isolated an additional allele, termed *p-ww2,* that specifies colorless kernel pericarp and cob (Figure 1A). The *p-ww2* allele was derived via an alternative transposition reaction involving *fAc* and a nearby, directly oriented *Ac* element inserted 3′ of *fAc* [4]*,* followed by excision of the *Ac* element. The structure of *p-ww2* is very similar to that of the four fusion alleles, except that *fAc* is joined to a site in exon 3 of *p2,* instead of intron 2 of *p2* (Figure 1C). Although the deletion in *p-ww2* is slightly smaller than those of the *P-oo* alleles, the colorless kernel pericarp and cob phenotype indicates that the *p* gene is not functional in *p-ww2,* whereas the four *P-oo* alleles that specify orange pericarp color indicate that the *p2/ p1* fusion genes are functional.

**Figure 3:**
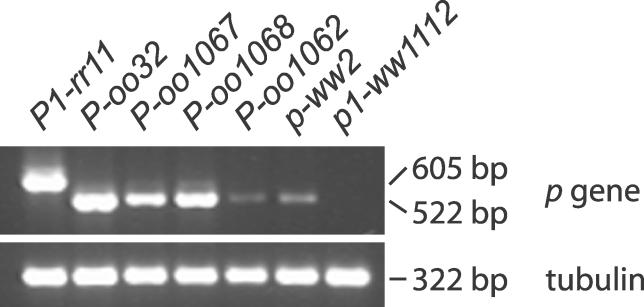
RT-PCR Analysis of *P-oo* Transcripts RNA was extracted from kernel pericarp (20 DAP), reverse transcribed, and PCR-amplified using primers complementary to both *p1* and *p2* transcripts. The progenitor allele *(P1-rr11)* shows amplification of a 605-bp band from *p1.* The *p-ww2* and *P-oo* alleles show amplification of a 522-bp band characteristic of the 5′ region of the *p2* gene. The *p1-ww1112* allele has a deletion of *p1;* the native *p2* gene is intact in this allele, but is not expressed in kernel pericarp.

To test for expression of the fusion alleles, we performed RT-PCR on RNA extracted from developing kernel pericarp. Previous studies showed that *p1* is expressed in various floral organs including kernel pericarp, while the *p2* gene is expressed in other tissues including maize silk, but not in pericarp. The PCR primers 1 and 3 amplify a product of 605 bp from the *p1* gene, and 522 bp from *p2* gene, due to different lengths of the 5′ UTR of each gene [5]. The progenitor allele *P1-rr11* has both *p1* and *p2* genes intact, and was used as a positive control. It generates a product of 605 bp as expected for *p1* expression in kernel pericarp. The *p1-ww1112* allele was used as a negative control; it has a deletion of the *p1* coding sequence, but retains the sequences upstream of *p1,* including the *p2* gene [10]. As expected, no products were amplified from this allele. The *P-oo* alleles generated RT-PCR products of 522 bp, which is consistent with expression of the fusion genes that include a 5′ UTR derived from the *p2* gene (Figure 3). Sequencing of the RT-PCR products confirmed that the *P-oo* transcripts contained exon 1 and exon 2 of *p2,* and exon 3 of *p1,* as predicted by the gene structures. The chimeric *P-oo* genes would encode a protein identical to that encoded by the *p1* gene except for a change in the fourth amino acid residue [5,12].

No expression of the unrearranged *p2* gene was detected in either *p1-ww1112* or *P1-rr11*. This is consistent with previous reports, and supports the conclusion that the native *p2* gene is not expressed in kernel pericarp [5]. It is somewhat surprising that *p-ww2* and the *P-oo* genes, each of which contain the *p2* promoter, generate transcripts in kernel pericarp. It has previously been shown that sequences nearly identical to genomic fragment 15 of the *p1* gene form part of an enhancer located approximately 5 kb upstream of the *p1* transcription start site [13]. In *p-ww2* and the *P-oo* alleles, the *p1* fragment 15 is located at new positions ranging from 6.2 kb to 14.4 kb 3′ of the *p2* transcription start site. At these new sites, the fragment 15 sequence may enhance expression of the fusion genes in pericarp. This idea is consistent with the observation that the intensity of pericarp pigment specified by each *P-oo* allele is approximately correlated with the size of the deletion; i.e., alleles in which the fragment 15 sequence is located closer to the *p2* promoter produce more intense pericarp color. Further analysis will be required to test this model.

## Discussion

Our results document four cases of exon shuffling induced by members of the *hAT* superfamily of DNA transposons. *hAT* elements are widespread in plants, animals, and fungi. The somatic rearrangement of vertebrate immunoglobulin genes through V(D)J recombination is catalyzed by proteins (Rag1/Rag2) that are functionally related to *hAT* family transposases [14,15]. Indeed, the formation of the *P-oo* alleles described here through transposase-induced intra-chromosomal deletion is analogous to the mechanism of vertebrate antibody gene rearrangement [16,17]. In contrast to the situation in vertebrates in which the immunoglobulin rearrangements are limited to somatic cells, the genome rearrangements detected in maize can be inherited because of the late recruitment of gametophytic cells during plant development [18].

Recent sequence analysis of the rice and maize genomes have shown that the Mutator and Helitron transposon families are involved in large-scale duplication and shuffling of coding sequences [19–21]. Although it is not yet known whether the resulting chimeric genes are functional, their sheer abundance suggests that these transposon-induced rearrangements could be an additional large potential source of chimeric genes.

Previous reports of exon shuffling in cultured human cells have been associated with illegitimate recombination, or retrotransposition of long interspersed nuclear elements [22,23]. Exon shuffling via retrotransposition can occur only when retroelements are inserted in or near exon sequences. In rice, the *Tos17* retrotransposon inserts preferentially into low-copy-number sequences [24]. In contrast, the vast majority of retroelement sequences in the maize genome are located predominantly in intergenic regions [25] and hence would not be expected to contribute to exon shuffling, whereas the tendency of *Ac* to insert preferentially into genic regions [26] greatly enhances its potential role in mediating exon shuffling reactions. Some cases of exon shuffling may confer a positive selective advantage that could promote fixation of variant chromosomal structures, such as inversions or reciprocal translocations, in sympatric populations [27,28].

Chromosomal rearrangements have been reported for other, non-*hAT,* transposon systems. In the fungus *Fusarium,* transposition involving termini of different *Tc1-mariner* elements can generate deletions and inversions that also may shuffle coding and regulatory sequences [29]. In *Drosophila,* transposition of *Foldback* elements and an associated *white* gene can result in activation of *white* gene expression, although little is known about the mechanism of *Foldback* transposition [30]. Also in *Drosophila,* transposition involving the termini of different *P* elements can induce various chromosomal rearrangements including deletions and inversions [31,32]. It seems likely that alternative transposition reactions of the type we report here are not unique to the *hAT* transposon superfamily, but may be a common feature of “cut-and-paste” eukaryotic transposons. Some transposable elements, such as *Ac/Ds* and *Sleeping Beauty,* tend to transpose to linked sites [33,34], leading to transposon clusters in which the termini of the linked transposons could be in either direct or reversed orientation. Alternative transposition reactions may then act upon these clustered transposon termini to generate large-scale chromosomal rearrangements. In support of this idea, a recent report has demonstrated that transgenic mice containing clusters of *Sleeping Beauty* transposon ends exhibit a high frequency of chromosomal aberrations [35]. Given the abundance of tandemly duplicated segments in plant and animal genomes, our results suggest that the alternative transposition events could represent an important evolutionary mechanism for the generation of new genes.

## Materials and Methods

### Genetic stocks.

Alleles of the maize *p1* gene are identified by a two-letter suffix that indicates their expression pattern in pericarp and cob: e.g., *P1-rr* (red pericarp and red cob); and *p1-ww* (white pericarp and white cob). The *P-oo* (orange pericarp and orange cob) alleles described here were derived from *P1-rr11* [3]; *p-ww2* was derived from *p1-vv9D9A* [4].

### Genomic DNA extractions and Southern blot hybridization.

Total genomic DNA was prepared from leaf tissue using a modified cetyltrimethylammonium bromide (CTAB) extraction protocol [36]. Agarose gel electrophoresis and Southern hybridizations were performed as described [37], except hybridization buffers contained 250 mM NaHPO_4_ (pH 7.2), 7% SDS, and wash buffers contained 20 mM NaHPO_4_ (pH 7.2), 1% SDS.

### PCR amplifications.

PCR amplifications were performed as described [38] using the following oligonucleotide primers: CGCGACCAGCTGCTARCCGTG, CCAAGGAGGAAGAAGA CATCATCATCAAG, GCAGCTTGCTCATGTCGATGGC, and GCAGCTTGCTCATGTCG ATGGC. HotMaster Taq polymerase from Eppendorf (Hamburg, Germany) was used in the PCR reaction. Reactions were heated at 94 °C for 3 min, and then cycled 35 times at 94 °C for 20 s, 63 °C for 30 s, and 65 °C for 1 min per 1 kb length of expected PCR product, then 65 °C for 8 min. In most of the PCR reactions 2 M betaine and 4%–8% DMSO were added. The band amplified was purified from an agarose gel and sequenced directly. Sequencing was done by the DNA Synthesis and Sequencing Facility, Iowa State University, Ames, Iowa, United States.

For RT-PCR, total RNA was extracted from 20 DAP (days after pollination) pericarp using the RNeasy plant mini kit by Qiagen (Valencia, California, United States of America) and treated with DNase (Qiagen) to remove residual genomic DNA. Using StrataScript Reverse Transcriptase by Stratagene (La Jolla, California, United States of America) with oligo(dT) at 42 °C, 1 μg of total RNA was reverse-transcribed, while 3 μl of cDNA was subject to PCR amplification.

Oligonucleotide primers used for screening the *P-oo* alleles by genomic PCR were: p2-5044f: CCAAGGAGGAAGAAGACATCATCATCAAG, p2-5802f: ATAATGTTCCTTACTTACAACCAGCGG, p2-6841f: AACAGTCCCGATGATGCCCGCCAC, p2-7438f: TACACACAAACACCTTCCACTCCATAAAAT, p2-7951f: CTGAAGAAATCCTCTAACAAAACTGGCG, p2-8208f: TCTGGTCCAACACTCCCTCTTCATTC, and p2-8658f: GCGATCAATGAGTGAGCTAGTTTGTTGC.

## Supporting Information

Video S1 Animation of Alternative Transposition Model for Generation of Chimerical Genes Press buttons to play animation. For details, see Figure 2 legend.
(34 KB MOV)

### Accession Numbers

The GenBank (http://www.ncbi.nlm.nih.gov/Genbank) accession numbers for *p1* and *p2* are z11879 and af210616, respectively.

## Abbreviations

bp: base pair
kb: kilobase

## References

[pgen-0020164-b001] Kunze R, Weil CF, Craig NL, Craigie R, Gellert M, Lambowitz AM (2002). The *hAT* and *CACTA* superfamilies of plant transposons. Mobile DNA II.

[pgen-0020164-b002] Coupland G, Plum C, Chatterjee S, Post A, Starlinger P (1989). Sequences near the termini are required for transposition of the maize transposon *Ac* in transgenic tobacco plants. Proc Natl Acad Sci U S A.

[pgen-0020164-b003] Zhang J, Peterson T (2004). Transposition of reversed *Ac* element ends generates chromosome rearrangements in maize. Genetics.

[pgen-0020164-b004] Zhang J, Peterson T (1999). Genome rearrangements by nonlinear transposons in maize. Genetics.

[pgen-0020164-b005] Zhang P, Chopra S, Peterson T (2000). A segmental duplication generated differentially expressed Myb-homologous genes in maize. The Plant Cell.

[pgen-0020164-b006] Grotewold E, Athma P, Peterson T (1991). Alternatively spliced products of the maize P gene encode proteins with homology to the DNA binding domain of Myb-like transcription factors. Proc Natl Acad Sci U S A.

[pgen-0020164-b007] Zhang J, Peterson T (2005). A segmental deletion series generated by sister-chromatid transposition of *Ac* transposable elements in maize. Genetics.

[pgen-0020164-b008] Zhang P, Wang Y, Zhang J, Snook M, Peterson T (2003). A maize QTL for silk maysin levels contains duplicated myb-homologus genes which jointly regulate flavone biosynthesis. Plant Mol Biol.

[pgen-0020164-b009] Page DR, Kohler C, da Costa-Nunes JA, Baroux C, Moore JM (2004). Intrachromosomal excision of a hybrid *Ds* element induces large genomic deletions in *Arabidopsis*. Proc Natl Acad Sci U S A.

[pgen-0020164-b010] Athma P, Peterson T (1991). *Ac* induces homologous recombination at the maize *P* locus. Genetics.

[pgen-0020164-b011] Xiao YL, Li X, Peterson T (2000). *Ac* insertion site affects the frequency of transposon-induced homologous recombination at the maize *p1* locus. Genetics.

[pgen-0020164-b012] Athma P, Grotewold E, Peterson T (1992). Insertional mutagenesis of the maize *P* gene by intragenic transposition of *Ac*. Genetics.

[pgen-0020164-b013] Sidorenko L, Li X, Tagliani L, Bowen B, Peterson T (1999). Characterization of the regulatory elements of the maize *P-rr* gene by transient expression assays. Plant Mol Biol.

[pgen-0020164-b014] Jones JM, Gellert M (2004). The taming of a transposon: V(D)J recombination and the immune system. Immunol Rev.

[pgen-0020164-b015] Zhou L, Mitra R, Atkinson PW, Hickman AB, Dyda F (2004). Transposition of *hAT* elements links transposable elements and V(D)J recombination. Nature.

[pgen-0020164-b016] Hiom K, Melek M, Gellert M (1998). DNA transposition by the RAG1 and RAG2 proteins: A possible source of oncogenic translocations. Cell.

[pgen-0020164-b017] Agrawal A, Eastman QM, Schatz DG (1998). Transposition mediated by RAG1 and RAG2 and its implications for the evolution of the immune system. Nature.

[pgen-0020164-b018] Walbot V, Evans MM (2003). Unique features of the plant life cycle and their consequences. Nat Rev Genet.

[pgen-0020164-b019] Jiang N, Bao Z, Zhang X, Eddy SR, Wessler SR (2004). Pack-MULE transposable elements mediate gene evolution in plants. Nature.

[pgen-0020164-b020] Morgante M, Brunner S, Pea G, Fengler K, Zuccolo A (2005). Gene duplication and exon shuffling by helitron-like transposons generate intraspecies diversity in maize. Nat Genet.

[pgen-0020164-b021] Bennetzen JL (2005). Transposable elements, gene creation and genome rearrangement in flowering plants. Curr Opin Genet Dev.

[pgen-0020164-b022] Moran JV, DeBerardinis RJ, Kazazian HH (1999). Exon shuffling by L1 retrotransposition. Science.

[pgen-0020164-b023] van Rijk AA, de Jong WW, Bloemendal H (1999). Exon shuffling mimicked in cell culture. Proc Natl Acad Sci U S A.

[pgen-0020164-b024] Yamazaki M, Tsugawa H, Miyao A, Yano M, Wu J (2001). The rice retrotransposon Tos17 prefers low-copy-number sequences as integration targets. Mol Genet Genomics.

[pgen-0020164-b025] SanMiguel P, Tikhonov A, Jin YK, Motchoulskaia N, Zakharov D (1996). Nested retrotransposons in the intergenic regions of the maize genome. Science.

[pgen-0020164-b026] Cowperthwaite M, Park W, Xu Z, Yan X, Maurais SC (2002). Use of the transposon *Ac* as a gene-searching engine in the maize genome. Plant Cell.

[pgen-0020164-b027] Rieseberg LH (2001). Chromosomal rearrangements and speciation. Trends Ecol Evol.

[pgen-0020164-b028] Turelli M, Barton NH, Coyne JA (2001). Theory and speciation. Trends Ecol Evol.

[pgen-0020164-b029] Hua-Van A, Langin T, Daboussi MJ (2002). Aberrant transposition of a Tc1-mariner element, impala, in the fungus Fusarium oxysporum. Mol Genet Genomics.

[pgen-0020164-b030] Moschetti R, Marsano RM, Barsanti P, Caggese C, Caizzi R (2004). FB elements can promote exon shuffling: A promoter-less white allele can be reactivated by FB mediated transposition in Drosophila melanogaster. Mol Genet Genomics.

[pgen-0020164-b031] Gray YH, Tanaka MM, Sved JA (1996). P-element-induced recombination in Drosophila melanogaster: Hybrid element insertion. Genetics.

[pgen-0020164-b032] Preston CR, Sved JA, Engels WR (1996). P-element-induced male recombination and gene conversion in Drosophila. Genetics.

[pgen-0020164-b033] Dupuy AJ, Akagi K, Largaespada DA, Copeland NG, Jenkins NA (2005). Mammalian mutagenesis using a highly mobile somatic Sleeping Beauty transposon system. Nature.

[pgen-0020164-b034] Fischer SE, Wienholds E, Plasterk RH (2001). Regulated transposition of a fish transposon in the mouse germ line. Proc Natl Acad Sci U S A.

[pgen-0020164-b035] Geurts AM, Collier LS, Geurts JL, Oseth LL, Bell ML (2006). Gene mutations and genomic rearrangements in the mouse as a result of transposon mobilization from chromosomal concatemers. PLoS Genet.

[pgen-0020164-b036] Saghai-Maroof MA, Soliman KM, Jorgensen RA, Allard RW (1984). Ribosomal DNA spacer-length polymorphisms in barley: Mendelian inheritance, chromosomal location, and population dynamics. Proc Natl Acad Sci U S A.

[pgen-0020164-b037] Sambrook J, Fritsch EF, Maniatis T (1989). Molecular cloning: A laboratory manual.

[pgen-0020164-b038] Saiki RK, Erlich HA (1989). The design and optimization of the PCR. PCR technology.

